# Molecular identification of adenovirus causing respiratory tract infection in pediatric patients at the University of Malaya Medical Center

**DOI:** 10.1186/1471-2431-10-46

**Published:** 2010-07-02

**Authors:** Juraina Abd-Jamil, Boon-Teong Teoh, Eddy H Hassan, Nuruliza Roslan, Sazaly AbuBakar

**Affiliations:** 1 Tropical Infectious Diseases Research and Education Center (TIDREC), Department of Medical Microbiology, Faculty of Medicine, University of Malaya, 50603 Kuala Lumpur, Malaysia

## Abstract

**Background:**

There are at least 51 adenovirus serotypes (AdV) known to cause human infections. The prevalence of the different human AdV (HAdV) serotypes varies among different regions. Presently, there are no reports of the prevalent HAdV types found in Malaysia. The present study was undertaken to identify the HAdV types associated primarily with respiratory tract infections (RTI) of young children in Malaysia.

**Methods:**

Archived HAdV isolates from pediatric patients with RTI seen at the University of Malaya Medical Center (UMMC), Kuala Lumpur, Malaysia from 1999 to 2005 were used. Virus isolates were inoculated into cell culture and DNA was extracted when cells showed significant cytopathic effects. AdV partial hexon gene was amplified and the sequences together with other known HAdV hexon gene sequences were used to build phylogenetic trees. Identification of HAdV types found among young children in Malaysia was inferred from the phylograms.

**Results:**

At least 2,583 pediatric patients with RTI sought consultation and treatment at the UMMC from 1999 to 2005. Among these patients, 48 (< 2%) were positive for HAdV infections. Twenty-seven isolates were recovered and used for the present study. Nineteen of the 27 (~70%) isolates belonged to HAdV species C (HAdV-C) and six (~22%) were of HAdV species B (HAdV-B). Among the HAdV-C species, 14 (~74%) of them were identified as HAdV type 1 (HAdV-1) and HAdV type 2 (HAdV-2), and among the HAdV-B species, HAdV type 3 (HAdV-3) was the most common serotype identified. HAdV-C species also was isolated from throat and rectal swabs of children with hand, foot, and mouth disease (HFMD). Two isolates were identified as corresponding to HAdV-F species from a child with HFMD and a patient with intestinal obstruction.

**Conclusions:**

HAdV-1 and HAdV-2 were the most common HAdV isolated from pediatric patients who sought treatment for RTI at the UMMC from 1999 to 2005. HAdV-B, mainly HAdV-3, was recovered from ~22% of the patients. These findings provide a benchmark for future studies on the prevalence and epidemiology of HAdV types in Malaysia and in the region.

## Background

Respiratory tract infections (RTI) are common in adults and children worldwide. The disease varies in severity, presenting as uncomplicated, subacute, acute and chronic infection. RTIs can be life threatening depending on the causative agent and host condition. In children, a high incidence of RTI is caused either by: 1) heightened exposure of young children to RTI infectious agents from siblings, friends, and child care; 2) environmental factors; and 3) inherited disorders of the immune system [1]. In industrialized and developed countries, nearly 50% of pediatric consultations are RTI related [2,3], and at least 1.9 million children died from acute RTI with 70% of them in Africa and Asia in 2000 [4].

There are a number of infectious agents that cause RTI. Bacteria, such as *Haemophilus influenzae*, *Streptococcus pneumoniae*, *Escherichia coli*, *Klebsiella pneumoniae*, *Mycoplasma pneumonia*, and *Chlamydia trachomatis *are among the most common. However, 80%-90% RTI are caused by viruses, such as respiratory syncytial virus (RSV), influenza virus, parainfluenza virus, and adenovirus (AdV) [1,5]. RSV is one of the most common agents of RTI in infants and young children in many countries. It is estimated to cause ~39% of all pneumonia cases and up to 6% of pneumonia-associated deaths [6]. Parainfluenza virus and influenza virus also are commonly isolated viruses from children with viral RTI.

HAdV-associated RTI is reportedly low. It accounts for 4%-10% cases of pneumonia, 2%-10% cases of bronchiolitis, and 3%-9% cases of croup [7]. Severity of infection associated with HAdV varies with the different HAdV serotypes [7-9]. There are at least 51 immunologically distinct HAdV types classified into six species, designated A to F [10]. Viruses causing RTI are usually isolated in the laboratory from patients' nasal secretions and serotyped by immunological typing methods [11-13]. In recent years, molecular identification of the virus has become more common, where distinction of the virus types can be made through specific genomic sequence amplification by polymerase chain reaction (PCR) and determination of the partial hexon gene sequence [14,15]. Presently, there is no report of the prevalent HAdV types causing infections among Malaysians. This could be due to the low infection and fatality rates of the infection resulting in limited interest in typing the virus. The present study was undertaken to type HAdV of pediatric patients younger than 5 years seen at the University of Malaya Medical Center (UMMC), Kuala Lumpur, Malaysia.

## Methods

### Virus

Twenty-seven archived HAdV isolates were recovered from the UMMC virology repository and used for the study. The isolates were derived mainly from the nasopharyngeal secretions (NPS) of children younger than 5 years diagnosed with RTI (Table 1). Virus isolation and propagation were performed using African green monkey kidney cells (Vero), human lung carcinoma cells (A549), and dog kidney cells (MDCK). The presence of HAdV was detected by immunofluorescence staining using specific antibodies (Cat. No. 5000; Light Diagnostics Inc., Salt Lake City, UT, USA) following manifestation of cytopathic effects. Virus inoculum was prepared and kept at -70°C until needed for genomic typing. Further maintenance and propagation of the HAdV isolates were performed in A549 cells.

**Table 1:** Human adenoviruses isolated at the University of Malaya Medical Centre from 1999 to 2005.

Sample	Year	Isolation site^a^	Age	Diagnosis	Serotype
AD01MY99	1999	NPS	NA^b^	Pneumonia	B3

AD02MY99	1999	NPS	3 mth	Chronic lung diseases	B7

AD04MY00	2000	RS	7 mth	HFMD	F40

AD05MY00	2000	TS	1 yr	HFMD	C2

AD06MY00	2000	TS	8 mth	HFMD	C1

AD07MY01	2001	RS	6 mth	Intestinal obstruction	F41

AD08MY00	2000	RS	NA	HFMD	C5

AD09MY01	2001	NPS	I yr	Acute bronchiolitis	C1

AD10MY01	2001	NPA	1 yr	Bronchiolitis	C2

AD11MY02	2002	NPS	1 yr	Bronchopnuemonia, hypochronic anemia	C1

AD12MY02	2002	NPS	1 yr	Bronchiolitis	C1

AD13MY03	2003	NPS	10 mth	Acute gastroenteritis	C2

AD14MY03	2003	NPS	1 yr	Viral fever	C5

AD15MY03	2003	NPS	8 mth	Acute bronchiolitis	C2

AD16MY03	2003	NPS	NA	Bronchopneumonia	C2

AD17MY04	2004	NPS	2 yr	Acute bronchiolitis	C1

AD18MY04	2004	NPS	9 mth	Pneumonia	C5

AD19MY04	2004	NPS	5 mth	Bronchiolitis	B3

AD20MY04	2004	NPS	2 yr	Pneumonia	C2

AD21MY04	2004	NPS	3 yr	Pneumonia	C1

AD22MY04	2004	NPS	1 yr	Bronchopneumonia	C2

AD23MY04	2004	NPS	5 mth	Bronchiolitis	C6

AD24MY04	2004	NPS	3 yr	Viral fever	B3

AD25MY04	2004	NPS	8 mth	Pulmonary collapse	C5

AD26MY04	2004	NPS	1 yr	Pneumonia	B3

AD27MY04	2004	NPS	3 mth	Acute bronchiolitis	C2

AD28MY05	2005	NPS	2 yr	Bronchopneumonia	B3

^a^NPS is nasopharyngeal secretion; RS is rectal swab; TS is throat swab

^b^Not available

### PCR amplification and genome sequencing

Viral genomic DNA was extracted using Tri Reagent^® ^(Molecular Research Center Inc., Cincinnati, OH, USA) following the manufacturer's protocol. The partial hexon gene was amplified using the primer pair, AdTU7 (5'-GCCACCTTCTTCCCCATGGC-3') and AdTU4 (5'-GTAGCGTTGCCGGCCGAGAA-3') to amplify a 1,001 bp fragment of the partial hexon gene (position 20,733 - 21,734; Accession # NC_001405). A nested polymerase chain reaction (PCR) also was performed on the amplified fragment using the primers, AdU-S (5'-TTCCCCATGGCNCACAACAC-3') and AdU-A (5'-GCCTCGATGACGCCGCGGTG-3') which resulted in a 956 bp fragment. Amplification was performed for 36 cycles consisting of a denaturation step at 94°C for 1 min, an annealing step at 50°C for 1 min, and an extension step at 72°C for 2 min. The extension was continued at 72°C for 7 min. The amplified DNA fragment was separated in 1.5% agarose gel (Promega, Madison, WI, USA), and purified using QIA Quick gel extraction system (Qiagen GmbH, Hilden, Germany) according to the manufacturer's protocol. The DNA fragments were sequenced at Macrogen Inc. (Seoul, Korea).

### Sequence and phylogenetic analysis

The partial hexon gene sequences were aligned and phylogenetic trees were drawn as previously described [16]. Briefly, the HAdV partial hexon gene sequences were analyzed using Sequencher 4.6 (Gene Codes Corporation, Ann Arbor, MI) and aligned against other available AdV sequences using ClustalX [17]. Phylogenetic trees were drawn using the maximum-likelihood method as implemented in PHYLIP 3.67 [18] and the maximum-parsimony method using MEGA4 [19]. Bootstrap values were obtained from a random sampling of 100 replicates. Reference HAdV sequences used to build the phylogenetic trees were obtained from the GenBank. Details on the reference sequences are shown in Table 2.

**Table 2:** Reference adenovirus (AdV) strains from the Genbank used for the typing of human AdV isolated at the University of Malaya Medical Centre.

Accession Number	Serotype	Country/Strain
HAdV_A_NC_001460	A12	ATCC

HAdV_B_NC_004001	B11	Slobitski

HAdV_C_NC_001405	C2	NA^a^

HAdV_D_NC_002067	D17	NA

HAdV_E_NC_003266	E4	Vaccine CL68578

HAdV_F_NC_001454	F40	Dugan

HAdV_F_L19443	F40	Dugan

HAdV_3_AY878716	B3	Guangzhou, China

HAdV_3_AF542108	B3	Korea

HAdV_3_AF542129	B3	Korea

HAdV_3_DQ086466	B3	NA

HAdV_7_AF053085	B7	S-1058; Japan

HAdV_7_AY769945	B7	95-81; Korea

HAdV_7_AF515814	B7	China

HAdV_7_AC_000018	B7	NA

HAdV_7_AY769946	B7	Strain 99-95; Korea

HAdV_7_AB243009	B7	Kyoto, Japan

HAdV_11_AC_000015	B11	NA

HAdV_11_AF532578	B11	Slobitski

HAdV_11_AY163756	B11	Ad11p Slobitski

HAdV_14_AY803294	B14	De Wit/ATCC VR1091

HAdV_14_DQ149612	B14	NA

HAdV_16_X74662	B16	ATCC CH.79

HAdV_21_AB053166	B21	AV-1645

HAdV_34_AY737797	B34	Compton/ATCC VR716

HAdV_34_AB052911	B34	NA

HAdV_35_AC_000019	B35	Old world monkey strain

HAdV_35_AY128640	B35	Holden/ATCC VR718

HAdV_35_AY271307	B35	35p

HAdV_35_AB052912	B35	Japan

HAdV_50_DQ149643	B50	NA

HAdV_50_AY737798	B50	Wan/ATCC VR1502

HAdV_1_AC_000017	C1	P1 C124G1

HAdV_1_Y17244	C1	Ad71

HAdV_2_AC_000007	C2	NA

HAdV_2_AY224392	C2	Korea

HAdV_2_AF542118	C2	Korea

HAdV_2_AJ293905	C2	Germany

HAdV_2_AJ293901	C2	United Kingdom

HAdV_5_AF542130	C5	Korea

HAdV_5_AF542128	C5	Korea

HAdV_5_AF542124	C5	Korea

HAdV_5_AC_000008	C5	NA

HAdV_5_AF542109	C5	Korea

HAdV_6_DQ149613	C6	NA

HAdV_6_Y17245	C6	Ton66

HAdV_40_X51782	F40	Dugan

HAdV_41_X51783	F41	Tak

HAdV_41_D13781	F41	Tak

^a^Not Available

The study was approved by the University Malaya Medical Centre Ethics Committee (Approval #794.6).

## Results

A total of 2,583 pediatric patients with RTI were treated at the UMMC from 1999 to 2005. Of these patients, 48 (<2%) were positive for HAdV by either direct immunofluorescence staining of the patient's NPS, PCR amplification, or virus isolation. HAdV also was isolated from the throats of patients with HFMD. These HFMD patients usually did not present with RTI symptoms, but throat swabs were routinely collected in addition to rectal swabs.

**Figure 1:**
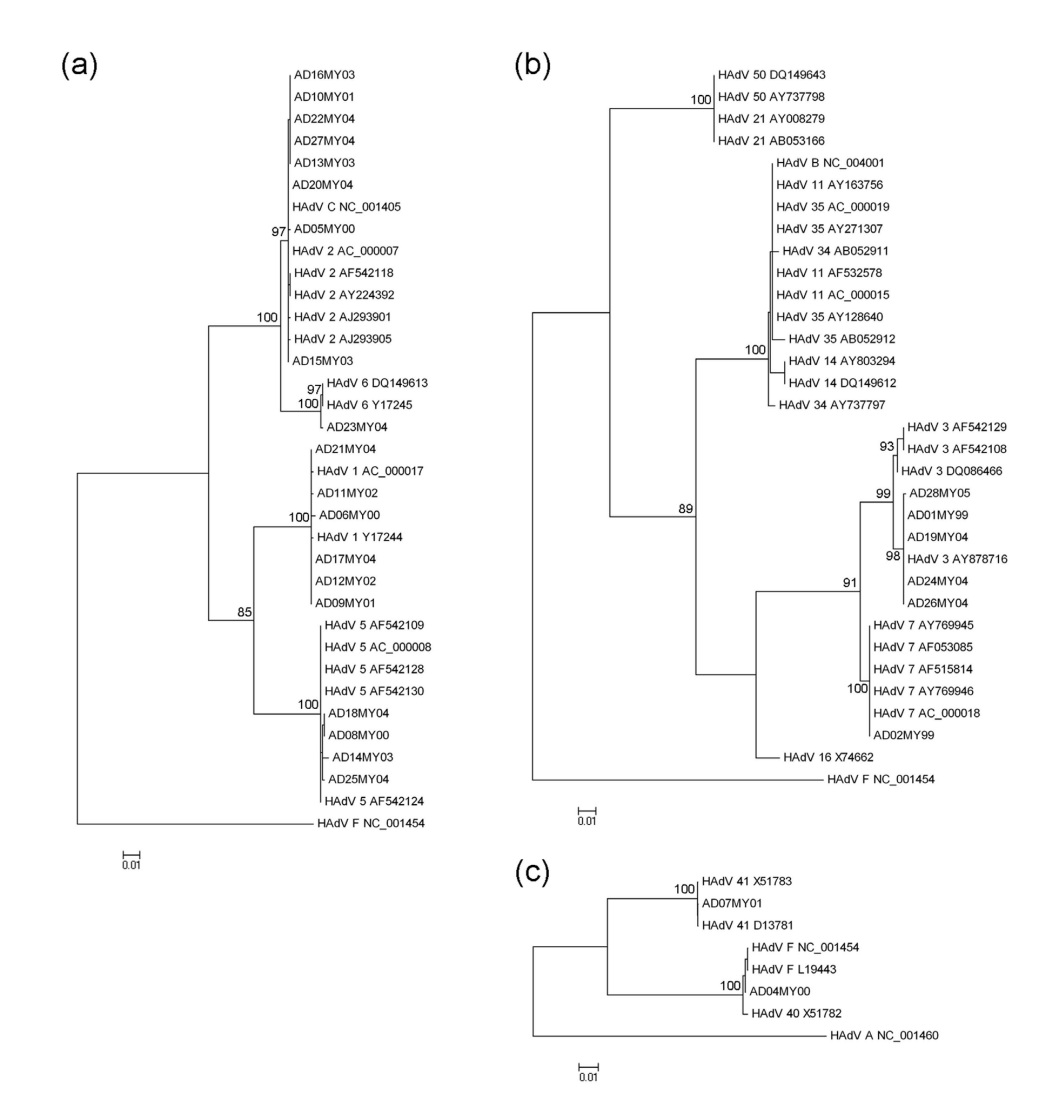
**Molecular typing of human adenoviruses (HAdV) isolated at the University of Malaya Medical Center (UMMC) from 1999 to 2005**. HAdV-1 and HAdV-2 comprised most of the HAdV-C species isolated at the UMMC (a). HAdV-B species consisted of HAdV-3 and HAdV-7. HAdV-3 formed its own distinct cluster separate from the rest of the group (b). Two isolates from the study were grouped as HAdV-F serotype 40 and 41 (c), commonly implicated in gastroenteritis.

In the present study, the partial hexon gene sequences (nucleotide 20,734-21,737) of 27 HAdV isolates from UMMC were determined. The sequences were aligned and phylogenetic trees were drawn using the maximum-likelihood and maximum-parsimony methods. Only results from the maximum-likelihood method were presented, as trees drawn from both the methods were similar. The nucleotide sequence alignment clustered the UMMC isolates into HAdV-C species (n = 19), HAdV-B species (n = 6), and HAdV-F species (n = 2) with high bootstrap values. Within the HAdV-C species, eight isolates were HAdV-2 (42%); six isolates, HAdV-1 (32%); four isolates, HAdV-5 (21%);, and one isolate, HAdV-6 (5%; Figure 1a). When compared with other HAdV from the GenBank, most of the UMMC HAdV-5 and HAdV-2 isolates formed a separate subcluster of their own (Figure 1a). Within HAdV-B species, five isolates grouped into HAdV-3, and one was HAdV-7 (Figure 1b). HAdV-3 isolates from the study formed a separate subcluster together with an isolate from Guangzhou, China (Accession # AY878716) distinct from other known HAdV-3. This raised the possibility that the viruses may share a common ancestral lineage with the Malaysian isolate that was isolated in 1999. The HAdV-7 clustered together with isolates from the East Asian countries suggesting a possible widespread presence of the virus in the East Asian region. Four of the HAdV isolates sequenced in the study were from HFMD patients, and three of them clustered together with the HAdV-C species. One isolate clustered with known HAdV-40, whereas an isolate from the rectal swab of a patient diagnosed with intestinal obstruction clustered with known HAdV-41. No HAdV-B genotype previously associated with fatal cases of HFMD [20,21] was detected in any of the HFMD patient samples. Isolates from HFMD patients were included in the study initially because no effort was made to discriminate the samples from those strictly with RTI. Patients with HFMD normally would not present with RTI symptoms.

## Discussion

Human adenovirus is most commonly associated with respiratory illnesses. However, depending on the infecting serotype, the virus also causes various other illnesses, including gastroenteritis, conjunctivitis, cystitis, and non- specific exanthemas [13,22]. Symptoms of the respiratory illness associated with HAdV range from mild infection to severe pneumonia [8,23]. Young children and immunocompromised patients are especially vulnerable to severe complications of HAdV infection [24,25]. The findings that less that 2% of UMMC pediatric RTI is associated with HAdV respiratory infection is consistent with other reports that HAdV-associated respiratory infection is usually low in comparison to other viruses, such as RSV and parainfluenza virus. The infection also is generally milder and rarely leads to severe complications and deaths [8,26]. The low number of HAdV isolation among pediatric patients seen at the UMMC also suggests that the virus is not associated with any major RTI outbreaks during the period from 1999 to 2005. This is perhaps among the reasons why there have not been many efforts to identify the HAdV species and types in children with RTI in many countries, including Malaysia. In addition, the low incidence of RTI caused by HAdV in the community hampered the effort to get enough representative isolates.

In our study, HAdV partial hexon gene sequences were used to type the different HAdV isolates. This gene region contains the hypervariable region that confers HAdV serotype specificity. Using this molecular typing method, HAdV-C species, specifically type 1 and 2, were the most common HAdV isolated from the pediatric patients seen at UMMC from 1999 to 2005. In contrast, studies done in the United States of America, United Kingdom, Korea, and China, showed HAdV-B species as the most commonly isolated HAdV [27-30]. The reasons for the marked differences are not known. It could be that HAdV-C is more common in the region in comparison to the more developed countries. However, the prevalence of HAdV-C species in the neighboring countries could not be compared because information from these countries are lacking.

Overrepresentation of HAdV-C in UMMC pediatric patients could suggest a high prevalence of the virus in the community. There are reports that the virus could persist and cause asymptomatic latent infection in rabbits for as long as one year [31]. HAdV-C serotypes 1, 2, and 5 are the most common serotype of HAdV associated in latent infection of tonsils and adenoids of humans, which at times cause RTI in young children [32]. The prolonged presence of the virus in infected children increases its transmissibility, and this could contribute to the persistence of the virus of young children in Malaysia. The ubiquitous presence of the virus also could help explain isolation of the virus from patients with HFMD and nonspecific viral fever. On the other hand, the results also could reflect the higher tendency of children with HAdV-C species infection to seek medical attention, hence suggesting that the virus could cause more severe manifestations of RTI. Further studies, however, will be needed to verify this.

## Conclusions

The present study is the first to report the prevalent and circulating HAdV types in Malaysia. It showed that HAdV-C species especially HAdV-1 and HAdV-2 were the most commonly isolated HAdV among pediatric patients seen at UMMC from 1999 to 2005, followed by HAdV-B species type 3. These viruses are common serotypes of HAdV causing acute RTI in pediatric patients. Because no such study has ever been reported in Malaysia, the present study provides a benchmark for future studies of HAdV infection in the country.

## Abbreviations

HAdV: human adenovirus; HFMD: hand, foot and mouth disease; NPS: nasopharyngeal secretion; RTI: respiratory tract infection; UMMC: University of Malaya Medical Center.

## Competing interests

The authors declare that they have no competing interests.

## Authors' contributions

SAB is the principal investigator of the study. SAB and JAJ designed the study and drafted and wrote the manuscript. NR and EHH propagated and maintained the virus isolates and performed the initial genomic sequence amplification. BTT performed the sequence alignment, sequence analysis, and tree-drawing. All authors have read and approved the final manuscript.

## Pre-publication history

The pre-publication history for this paper can be accessed here:

http://www.biomedcentral.com/1471-2431/10/46/prepub
